# Numerical Study of Tunable Photonic Nanojets Generated by Biocompatible Hydrogel Core-Shell Microspheres for Surface-Enhanced Raman Scattering Applications

**DOI:** 10.3390/polym11030431

**Published:** 2019-03-06

**Authors:** Yu-Jui Wang, Chi-An Dai, Jia-Han Li

**Affiliations:** 1 Department of Engineering Science and Ocean Engineering, National Taiwan University, No. 1, Sec. 4, Roosevelt Road, Taipei 10617, Taiwan; r06525100@ntu.edu.tw; 2 Department of Chemical Engineering, National Taiwan University, No. 1, Sec. 4, Roosevelt Road, Taipei 10617, Taiwan; polymer@ntu.edu.tw

**Keywords:** core-shell microsphere, photonic nanojet, polymer, biocompatible, surface-enhanced Raman scattering

## Abstract

Core-shell microspheres have been applied in various research areas and, in particular, they are used in the generation of photonic nanojets with suitable design for photonic applications. The photonic nanojet is a narrow and focused high-intensity light beam emitting from the shadow-side of microspheres with tunable effective length, thus enabling its applications in biosensing technology. In this paper, we numerically studied the photonic nanojets brought about from biocompatible hydrogel core-shell microspheres with different optical properties. It was found that the presence of the shell layer can significantly affect the characteristics of the photonic nanojets, such as the focal distance, intensity, effective length, and focal size. Generally speaking, the larger the core-shell microspheres, the longer the focal distance, the stronger the intensity, the longer the effective length, and the larger the focal size of the generated photonic nanojets are. The numerical simulations of the photonic nanojets from the biocompatible core-shell microspheres on a Klarite substrate, which is a classical surface-enhancing Raman scattering substrate, showed that the Raman signals in the case of adding the core-shell microspheres in the system can be further enhanced 23 times in water and 108 times in air as compared in the case in which no core-shell microspheres are present. Our study of using tunable photonic nanojets produced from the biocompatible hydrogel core-shell microspheres shows potential in future biosensing applications.

## 1. Introduction

Core-shell microspheres have been used for various applications, such as the removal of microcystins [1], magnetic separation of proteins [2], immobilization of trypsin [3], supercapacitors [4], and surface-enhanced Raman spectroscopy with photonic nanojets [5]. Among these different applications, the generated photonic nanojets by core-shell microspheres have been taken a lot of attentions in photonic applications [5]. The photonic nanojets are found to have the long-tail, non-resonant, and strong light intensity with long effective length, emanating from the shadow side of the illuminated objects. They can be generated by different shapes and microstructures of the objects, such as dielectric circular cylinders [6,7], microspheres [8,9], micro ellipsoids [10,11], micro cube [12], micro triangular body, micro hexahedron, micro triangular cone [13,14], micro disk [15], hemisphere [16], micro-toroid [17], and core-shell microspheres [18,19,20,21]. In order to create photonic nanojets with strong light intensity, the structural parameters and materials used in the illuminated and transparent objects need to be carefully chosen and suitably designed. The photonic nanojets can be affected by various factors, including the refractive indices of the materials being used, sizes, shapes, and the structure of the objects, incident light wavelength, surrounding medium, etc. The generated photonic nanojet often has a sub-wavelength and high-intensity focus spot with a long effective length. Thus, the photonic nanojets are applied in several optical and photonic applications, including super-resolution white-light nanoscopy [22], optical waveguide [23], enhancement of Raman scattering signals [24,25,26,27,28,29], optical nano-patterning [30], detection of single nanoparticle and biomolecules [31], enhancement of backscattering of light [32], optical data-storage [33], optical forces [34], optical super-resolution photoacoustic microscopy [35], laser surgery [36], and photon fluorescence enhancement [37].

Tuning photonic nanojets propagation distance is often the most important issue for many applications. For some applications, the mismatch of this propagation distance of the photonic nanojet with experimental setups may cause great difficulties if the photonic nanojets cannot be well tuned. In order to solve this problem, fabricating a shell layer with different refractive index on a homogenous microsphere to form a so-called core-shell microsphere, may give additional functionalities for tuning the photonic nanojets, including better controls of the propagation distance, intensity, focal size, etc. Many research results have demonstrated that the many properties of the core-shell micro-structures can be used to tune their resulting photonic nanojets, such as core-shell microsphere, core-shell-micro cylinder [38,39], core-shell-micro hemisphere [40], and core-shell-micro cube [41]. For example, Kong et al. demonstrated that the dielectric microspheres with graded-index can generate a significantly elongated photonic nanojet [18]. Gu et al. presented that a modern design and two-dimensional photonic nanojet worked over a distance of 100 times of the light wavelength by illuminating liquid-filled hollow micro-cylinder under liquid immersion [39]. Wu et al. reported that an ultra-stretched and three-dimensional photonic nanojet propagated a distance over 57 times of the wavelength of the incident light [20]. Furthermore, Wu et al. investigated that the full width at half maximum (FWHM) and effective length of the photonic nanojets can be modulated by changing the temperature of the vanadium oxide which is coated onto the top-half-surface of a glass microsphere [21]. Xie et al. proposed a two-layer dielectric hemisphere, which can be used to generate ultra-long photonic nanojets [40]. Bontempi et al. demonstrated that the SiO_2_/TiO_2_ core/shell microspheres can be used for application of plasmon-free surface-enhanced Raman spectroscopy [5]. As stated above, longer photonic nanojets can be acquired by suitably designing the core-shell microspheres. By modulating the core-shell microspheres, the properties of photonic nanojets, such as FWHM, effective length, focal distance, and light intensity, can be controlled for further experimental study. The results of core-shell microspheres show that the photonic nanojets can have narrow FWHM, strong intensity, and super-long propagating distance by designing the structures and choosing the materials carefully. However, the materials in most research are based on dielectric materials or homogeneous particles (e.g., core-only particles) immersed in liquids [20,39]. In addition, the fabrication processes for liquid-immersed core-shell dielectric microspheres are often complicated. Thus, the study of tunable photonic nanojets by core-shell microspheres is increasingly important. We studied the core-shell microspheres made from biocompatible hydrogel material. It has great potential for biosensing applications, not only because it has the merit of photonic nanojets, but also has the biocompatible advantages. To understand the properties of photonic nanojets generated by core-shell microspheres, we studied different structural parameters of the core-shell microspheres, including the effect of shell thickness, overall size of the core-shell microspheres, the surrounding environment, etc. It was found that the photonic nanojets can be tuned by changing the thickness of the shell layer, the environment, and overall sizes of the hybrid structures for the design of various practical experiments. To demonstrate the potential applications by using our designed core-shell microspheres, we applied this biocompatible hydrogel core-shell microsphere to enhance the surface-enhanced Raman scattering (SERS) signals on a Klarite substrate, which is one of the most popular commercially-used substrates for further enhancing the SERS technique.

## 2. Methodology

**Figure 1:**
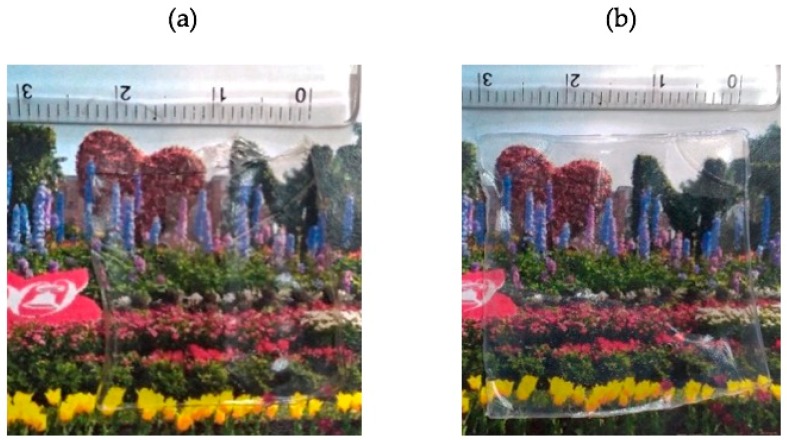
Photos of biocompatible hydrogel materials, (**a**) poloxamer 407 diacrylate (P407DA)/ polyacrylic acid (PAA) and (**b**) P407DA/poly (hydroxyethyl methacrylate) (PHEMA) placed in front of a photo picture.

Biocompatible hydrogel materials have been used for various biomedical applications [42,43,44]. It was found that some biocompatible hydrogel materials have high transmission property in the visible light region so that they can be used for the study to develop suitable material to construct artificial corneas [44]. In this paper, the materials we chose in designing the core-shell microspheres to generate the photonic nanojets were two interpenetrating network of poloxamer 407 diacrylate (P407DA)/ polyacrylic acid (PAA) and P407DA/poly (hydroxyethyl methacrylate) (PHEMA). P407DA is a commercially available triblock copolymer of polyethylene oxide-*b*-polypropylene oxide-*b*-polyethylene oxide with both chain ends capped with a double bond for subsequent crosslinking to form a hydrogel network. These three materials were chosen because they are all biocompatible hydrogel materials with good transmittance and high water content. Their high optical transparency can be demonstrated by placing a film made from them in front of an image with a photo picture shown in Figure 1. The optical properties of the P407DA/PAA and P407DA/PHEMA hydrogels depend on the concentrations of each components and fabrication procedures. The refractive indices of the constituting P407DA/PAA and P407DA/PHEMA hydrogels for the core-shell microspheres in this paper were measured to be around 1.3640 and 1.4232 at 589 nm of an incident light by using a refractometer (ATAGO ABBE T3).

The simulations of photonic nanojets generated by the core-shell microspheres were done by using Lumerical FDTD Solutions, a commercial software based on the finite-difference time-domain method. We set up the core-shell microspherical structures with the biocompatible hydrogel materials and used the transverse electric (TE) polarization for an incident light with a wavelength of 589 nm. The simulation domain was set up to be 8 μm × 8 μm × 107 μm. Uniform meshes were applied in the domain and the mesh spacing was determined to be about 30 nm to ensure that the simulation results can be numerically converged in a reasonable numerical errors. The perfectly matched layers (PML) were arranged around the boundaries for the study of photonic nanojets by using core-shell microspheres. 

**Figure 2:**
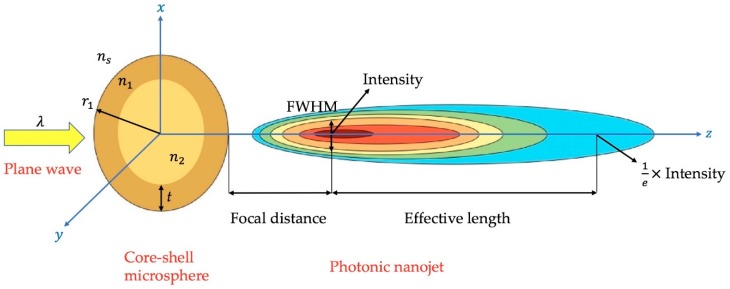
A schematic diagram of the produced photonic nanojet which is generated by a core-shell microsphere. λ is the incident light wavelength, $r_{1}$ is the radius of the overall core-shell microspheres, *t* is the thickness of the shell, $n_{1}$ and $n_{2}$ are the refractive indices of the shell and core, respectively, and $n_{s}$ is the refractive index of the surrounding environment. FWHM stands for full width at half maximum.

The refractive index of the material was one of the most important parameters for the study of the generation of photonic nanojets [45]. Figure 2 shows a schematic diagram of photonic nanojet which was produced by illuminating the core-shell microsphere. The formed elliptical photonic nanojet depends on the surrounding environment, refractive indices of core-shell microspheres, overall size of core-shell microspheres, and incident light wavelength. There are usually four important characteristics to represent the property of the resulting photonic nanojets, including the focal distance, intensity, effective length, and FWHM [20]. The focal distance presents the distance between the edge of the structure and the position of the maximum intensity of photonic nanojet on the *z* optical axis. The focal point location depends on the choice of the refractive indices of core-shell microspheres. The produced intensities shown in our simulation are the strongest electric field intensities in the focal spots of the photonic nanojets. The effective length means the distance from the strongest light intensity of the focal spot to the 1/*e* of the magnitude of the strongest light intensity behind the focus. The FWHM is the full width at half maximum of the focus, which represents the beam waist in the *x* axis. 

## 3. Results and Discussion

In this section, we discuss the simulation results of varying the structural parameters of the core-shell microspheres. The results included changing the thickness of shell layer, overall size of the core-shell microsphere, and surrounding environment. Additionally, we present the simulation which used the designed biocompatible core-shell microspheres for further improving the SERS signal.

### 3.1. Effects of the Thickness of the Shell of Core-shell Microspheres on Photonic Nanojets

**Figure 3:**
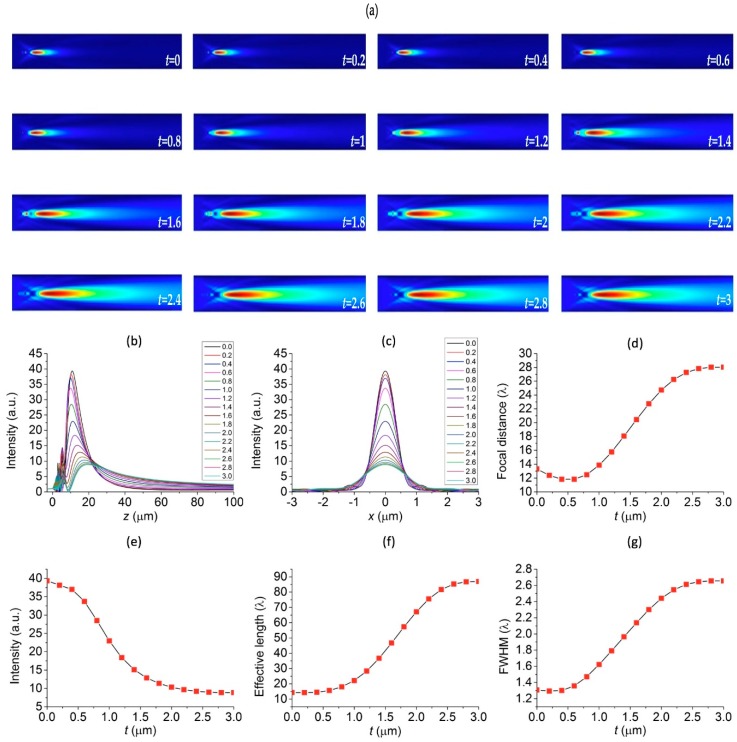
Visualizations of the FDTD-computed electric field intensity profiles by changing the shell thickness of the high-core core-shell microspheres. The thickness of the shell layer changes from 0 to 3 μm with an increment of 0.2 μm: (**a**) the electric field intensity profile, (**b**) electric field intensity along the *z* optical axis, (**c**) electric field intensity along the cross section direction at the focal spot, (**d**) focal distance, (**e**) intensity, (**f**) effective length, and (**g**) FWHM.

To study the effects of the thickness of the shell layer of the core-shell microspheres, *t*, on the properties of generated photonic nanojets, we varied the thickness of the shell *t* and fixed all other structural parameters, i.e., $r_{1}$ = 3 μm, λ = 589 nm, and $n_{s}$ = 1.33 (in water), respectively. The biocompatible hydrogel materials P407DA/PAA and P407DA/PHEMA, whose refractive indices are 1.3640 and 1.4232, respectively, were chosen for either the material of the core or shell layer in our simulations. We consider two cases in which the “high-core” case and “low-core” case stand for microspheres with higher and lower refractive indices, respectively, in the core region of the core-shell microspheres. Therefore, the high-core core-shell microsphere means that its core was composed of the higher refractive index P407DA/PHEMA, while the shell layer was composed of the lower refractive index P407DA/PAA. On the other hand, the low-core core-shell microsphere represents that the core consisted of the P407DA/PAA and its shell consisted of P407DA/PHEMA. In both cases, we changed the thickness of the shell layer, *t*, from 0 to 3 μm. The cases with shell thickness *t* = 0 μm and 3 μm were the cases of microspheres without the shells. Figure 3a shows that the electric field intensity profiles of photonic nanojets emanating from the high-core core-shell microspheres as a function of different thickness of the shell layer, *t*, ranging from 0 to 3 μm with an increment of 0.2 μm, where the electric field intensity was obtained by taking the calculated electric field magnitude to the second power. It can be seen that the photonic nanojets exhibit longer tails as the thickness of the shell layer increases. Figure 3b shows the corresponding electric field intensity plotted along the optical axis (*z* axis) for the high-core core-shell microspheres. This showed that the focal spots of photonic nanojets moved to a longer distance away from the microspheres and the intensity decreased as the thickness of the shell layer increased. Figure 3c shows the electric field intensity profile plotted along the cross sectional direction (*x* axis) at the focal spots for the high-core core-shell microspheres and shows that the focal spots became wider and that the intensity decreased as the thickness of the shell increased. To describe the optical properties of the photonic nanojets, four important characteristics, including focal distance, intensity, effective length, and FWHM are shown in Figure 3d–g. This shows that the focal distance increased, the intensity decreases, the effective length became longer, FWHM got wider as the thickness of shell layer increased. This was because the shell layer had a low refractive index with less refractive power as compared with high refractive-index material. 

**Figure 4:**
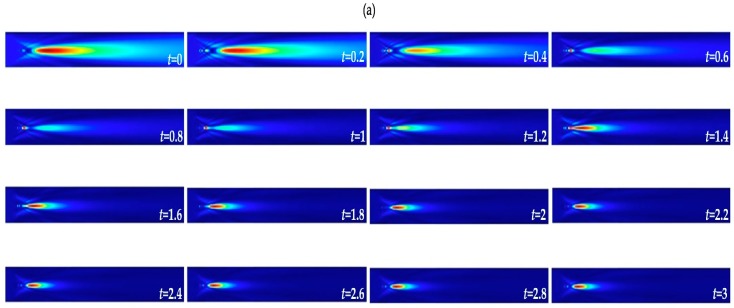

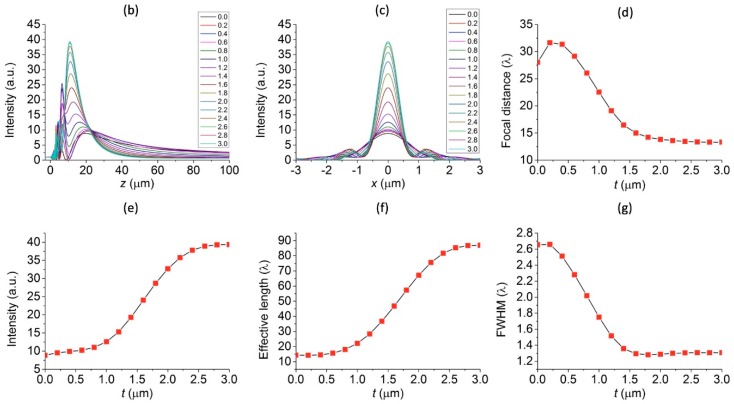
Visualizations of the FDTD-computed electric field intensity profiles by changing the shell thickness of the low-core core-shell microspheres. The thickness of the shell layer changes from 0 to 3 μm with an increment of 0.2 μm: (**a**) the electric field intensity profile, (**b**) electric field intensity along the *z* optical axis, (**c**) electric field intensity along the cross sectional direction at the focal spot, (**d**) focal distance, (**e**) intensity, (**f**) effective length, and (**g**) FWHM.

Correspondingly, Figure 4 shows the simulation results for the photonic nanojets profile for the low-core core-shell microspheres, in which the core consists of low refractive index P407DA/PAA and the shell consists of high refractive index P407DA/PHEMA. Figure 4a shows the electric field intensity profile in *x*-*z* plane of the produced photonic nanojets for the low-core core-shell microspheres with shell thickness ranging from 0 to 3 μm. Figure 4b,c show the propagation of the photonic nanojets along the *z* optical axis and the cross sectional *x*-axis direction of the electric field intensity of the maximum intensity by varying the shell thickness of the core-shell microspheres. In the low-core core-shell microsphere case, there was a dramatic transformation of the produced photonic nanojets for shell thickness of 0.4 μm, 0.6 μm, 0.8 μm, 1.0 μm, and 1.2 μm. In these five cases, the largest intensities were located in front of the photonic nanojets as shown in Figure 4a,b; moreover, it was found that the spot sizes of the largest intensities of these five cases were smaller than the FWHMs of the produced photonic nanojets. Notably, because of the higher refractive index for the shell layer, light bend more as it transmits through the shell layer as compared to the core material in the case of low-core core-shell microspheres. This results in smaller focal spots in front of the long-tailed photonic nanojets for some low-core core-shell microspheres. Figure 4d–g shows the focal distance, intensity, effective length, and FWHM for the low-core core-shell microspheres. They show that the focal distance became shorter, the intensity increased, and the effective length and FWHM decreased as the shell thickness increased. This was because the shell with high refractive index, such that the low-core core-shell microsphere had more refractive property as compared to the case in which high-core core-shell microspheres, was used. Comparing between Figure 3 and Figure 4, we can conclude that the photonic nanojets can be adjusted by varying the shell thickness in both high-core and low-core core-shell microspheres.

**Figure 5:**
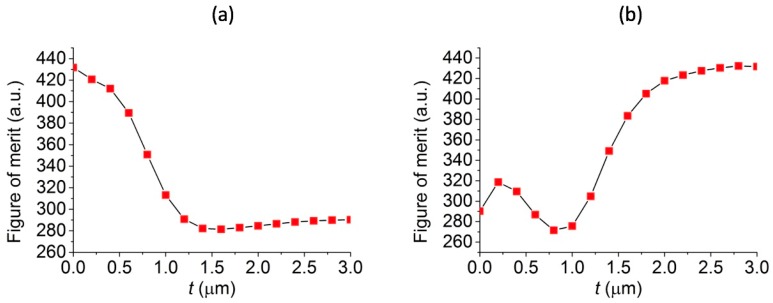
FOM for the core-shell microspheres in (**a**) high-core and (**b**) low-core cases.

A so-called figure of merit (FOM) to access the quality of photonic nanojet is proposed to quantify the characteristics of an intense light beam, and this FOM can be defined as (*L* × *I*)/*w* plotted as a function of *t*, where *L*, *I*, and *w* are the effective length, intensity, and FWHM of the photonic nanojet, respectively [18]. Therefore, it was assumed that the higher the FOM value is, the larger the intensity, the longer the effective length, and the narrower the spot size is. To understand the characteristics of photonic nanojets, which are generated by core-shell microspheres with different shell thickness, we used the FOM to examine these photonic nanojets. Figure 5a,b are the FOM plots for the high-core and low-core photonic nanojets based on the calculations obtained from the results shown in Figure 3 and Figure 4. Overall, it was found that for the case of low-core photonic nanojets, the microsphere with a shell layer thickness of 2.6, 2.8, and 3.0 μm had the saturated FOM values, which means that they had similar photonic characteristics. The case with a shell layer of 3.0 μm was the case of the microsphere with a higher refractive index. Although the microsphere with higher refractive index can have similar photonic characteristics as compared to the low-core cases with the shell layer thickness as 2.6 and 2.8 μm, the core-shell microspheres with shell layer thickness from 0 to 3 μm can have more functionality on the tunable focal distance and effective length with a preferable light intensity or focal size. For some applications, one of the four characteristics in Figure 3d–g and Figure 4d–g may be more important than the other three characteristics. For example, the photonic nanojets generated by the microspheres can be used to enhance the SERS signals [26,27], and the light intensity and focal distance of photonic nanojets are more important than the effective length and focal spot size. Thus, we think that the FOM may be useful to define the quality of photonic nanojets in some applications; however, it may need to consider the four characteristics of photonic nanojets for some other applications. Here, the four characteristics and FOM of photonic nanojets in Figure 3, Figure 4 and Figure 5 show the tunable ability of these core-shell microspheres. In other words, the ratio of a shell thickness to a radius of the whole core-shell microsphere of 13/15, 14/15, and 15/15 in the low-core core-shell microspheres may have larger FOM for the photonic nanojets developed. Therefore, this ratio was fixed and we studied the overall size effect of the microspheres on the properties of the photonic nanojets, as described in the next section.

### 3.2. Effects of Size of Core-Shell Microspheres on Photonic Nanojets

**Figure 6:**
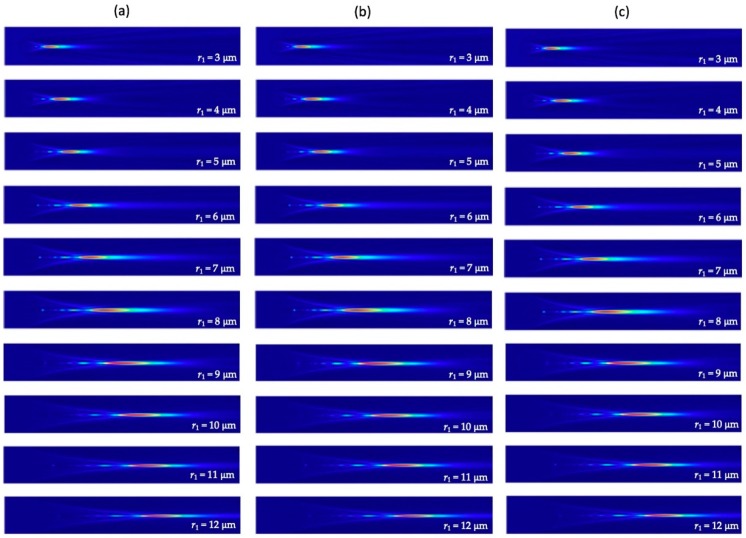
Visualizations of the FDTD-computed electric field intensity profiles of the produced photonic nanojets by varying $r_{1}$ of the core-shell microspheres from 3 μm to 12 μm in low-core cases: (**a**) $t/r_{1}$ = 3/15, (**b**) $t/r_{1}$ = 14/15, and (**c**) $t/r_{1}$ = 15/15.

**Figure 7:**
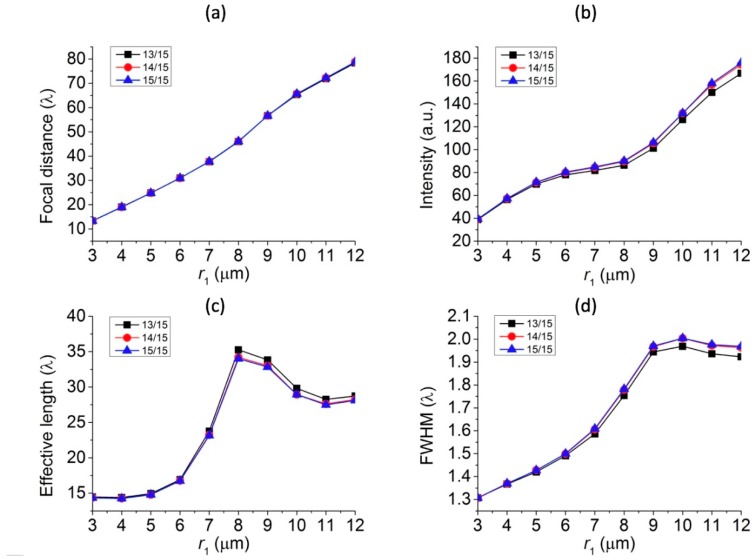
Four characteristics of photonic nanojets by varying the overall size, $r_{1}$ of the core-shell microspheres from 3 μm to 12 μm in low-core cases ($t/r_{1}$ = 13/15, 14/15, and 15/15): (**a**) focal distance, (**b**) intensity, (**c**) effective length, and (**d**) FWHM.

**Figure 8:**
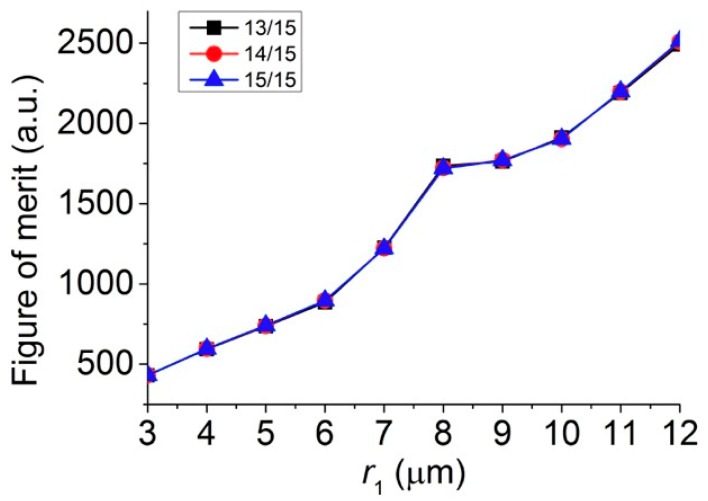
FOM of the core-shell microspheres by varying $r_{1}$ of the core-shell microspheres from 3 μm to 12 μm in the low-core cases ($t/r_{1}$ = 13/15, 14/15, and 15/15).

The overall size of core-shell microspheres is often considered to have the most significant influence on the intensity and focal distance of the photonic nanojets generated. It was found that there is essentially no photonic nanojets produced for very small spheres in the Rayleigh regime, i.e., the radius of spheres that is much smaller than the wavelength of the incident light [46]. However, in the Mie regime, in which the particle size was bigger than the wavelength of the incident light, the corresponding photonic nanojets can be efficiently generated. To study the overall size effect of the core-shell microspheres on the photonic nanojets produced, we considered the cases in which the three higher FOM values in Figure 5b were chosen and the following parameters were used; namely, $t/r_{1}$= 13/15, 14/15, and 15/15, λ = 589 nm $n_{1}$ = 1.4232 $n_{2}$ = 1.3640, and $n_{s}$ = 1.33 (in water). We varied the overall radius of the core-shell microspheres, $r_{1}$, from 3 to 12 μm by fixing the ratio of $t/r_{1}$ = 13/15, 14/15, and 15/15. The corresponding simulation results of the electric field intensity profile thus produced are shown in Figure 6. It can be seen that the tails of the photonic nanojets became longer as the overall size of the core-shell microspheres grew bigger. By extracting the simulation results shown in Figure 6, the variation of focal distance, intensity, effective length, and FWHM of the formed photonic nanojets are plotted in Figure 7. This shows that the focal spots of the photonic nanojets moved further away from the core-shell microsphere surface and the corresponding intensity increased as the size of the core-shell microsphere increased. This shows that the focal distance increased, the intensity got stronger, the effective length became longer, and the FWHM became wider as the overall size of the core-shell microspheres increased. Although the results for the ratio of $t/r_{1}$ = 13/15, 14/15, and 15/15 in Figure 6 look similar, they still have some minor differences for the four characteristics in Figure 7. For the case with a higher ratio of $t/r_{1}$, the intensity got larger, the effective length was smaller, and the FWHM was larger with similar focal distance as the size of core-shell microsphere increased. Figure 8 shows the FOM of the core-shell microspheres by varying $r_{1}$ of the core-shell microspheres from 3 μm to 12 μm in low-core cases ($t/r_{1}$=13/15, 14/15, and 15/15), and this shows that the FOM got larger as the size of the core-shell microsphere became larger. Although the FOMs in Figure 8 have the similar trends, the four photonic characteristics in Figure 7 still had some differences for these three shell thickness cases $t/r_{1}$= 13/15, 14/15, and 15/15. As discussed in Section 3.1, FOM may be used to define the quality of photonic nanojets in some applications; however, it may still need to consider the four characteristics of photonic nanojets for some other applications. The core-shell microspheres have more tunable ability as compared to pure microspheres. The scattering property of microsphere can be characterized by the refractive index of the microspheres and the size parameter (*q* = 2π*R*$n_{s}$/λ), where *R* is the radius of the particle, and the maximum intensity of the photonic nanojets by microspheres for the size in the scale of Mie scattering have an asymptotic dependence for large size parameters, i.e., $I_{max}\propto q$ [45]. The cases we consider in Figure 6, Figure 7 and Figure 8 are in the scale of Mie scattering. From the results shown in Figure 7, the variation of the intensity and effective length of the photonic nanojets are more significant than the variation of the FWHM of the photonic nanojets. Thus, the FOM in Figure 8 got larger as the core-shell microspheres became bigger. However, if the size of the microsphere gets increasingly larger, it approaches the geometrical optics and the whole concept of photonic characteristics of photonic nanojets follows by Snell’s law [45]. This means that the focal distance is Rn/2(n−1), the FWHM is about $R\sqrt{\frac{{\left(4-n^{2}\right)}^{3}}{27n^{4}}}$, and the intensity is $I\sim \frac{R^{2}}{FWHM^{2}}=\frac{27n^{4}}{{\left(4-n^{2}\right)}^{3}}$ [45], where *n* is the refractive index of the microsphere. In the limit of geometrical optics, the effective length is also related to the *R*. Thus, the FOM approaches to a constant in the limit of geometrical optics. According to the results in Figure 8, which is in the scale of Mie scattering, the larger core-shell microsphere has larger FOM for the radius $r_{1}$ from 3 to 12 μm. The radius of the core-shell microsphere with the largest FOM may be too large to predict by the calculations of FDTD simulation, and it can be estimated by theoretical series expansions from Mie scattering. Although a larger FOM may be better for some applications, we only considered the size of the core-shell microsphere in the range for the applications of SERS in this paper. In the next subsections, we considered the effects of surrounding medium and consider the core-shell microspheres with suitable size for the SERS applications.

### 3.3. Effects of Surrounding Medium on Photonic Nanojets

**Figure 9:**
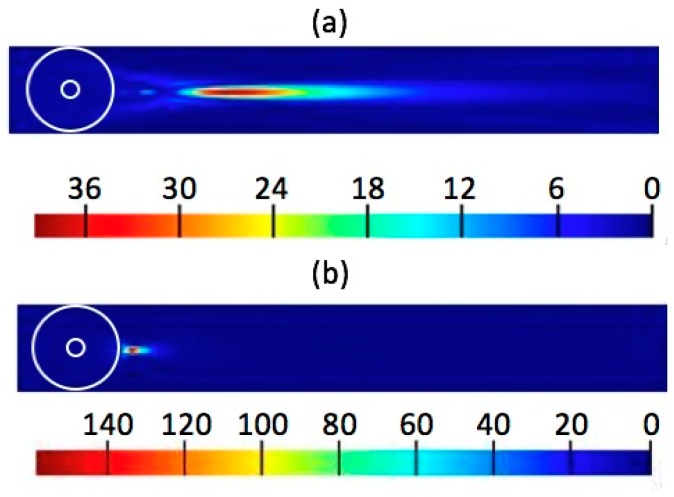
Visualizations of the FDTD-computed electric field intensity of photonic nanojets in different surrounding medium: (**a**) in water, (**b**) in air.

It was found that as the surrounding media changed, the characteristics of photonic nanojets changed significantly. To study the effect of surrounding medium around the core-shell microspheres on the photonic nanojets thus produced, we considered the case with $t/r_{1}$, λ, $n_{1}$, $n_{2}$, and *r*_1_ as 13/15, 589 nm, 1.4232, 1.3640, and 3 μm, respectively. We varied the surrounding medium in water ($n_{s}$ = 1.33) and air ($n_{s}$ = 1). The simulation results of the electric field intensity are shown in Figure 9. It can be seen that the tail of photonic nanojet in water was much longer than that in air environment. To describe the optical properties of the photonic nanojets, four important characteristics, including focal distance, intensity, effective length, and FWHM are shown in Table 1. Although the intensity of the photonic nanojet in water was smaller than that in air, it still had very strong enhancement with long effective length and focal distance. For this core-shell microsphere, the FOM in water was larger than the FOM in air.

### 3.4. Enhancements of SERS Signals by Core-Shell Microspheres

**Figure 10:**
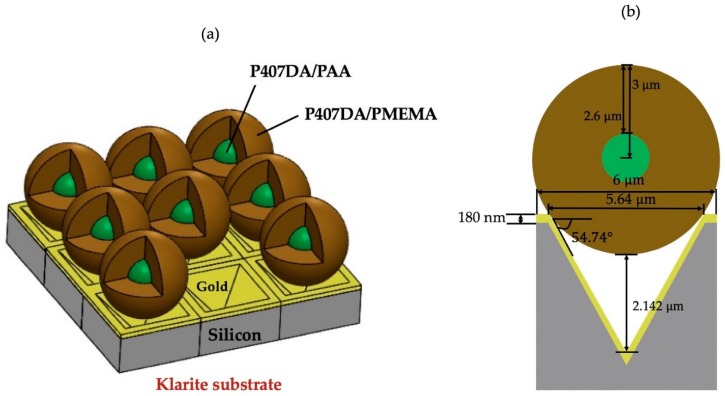
Schematic diagrams set up by core-shell microspheres on Klarite substrates for improving surface-enhanced Raman spectroscopy: (**a**) a periodic Klarite structure and (**b**) cross section of the unit structure.

**Figure 11:**
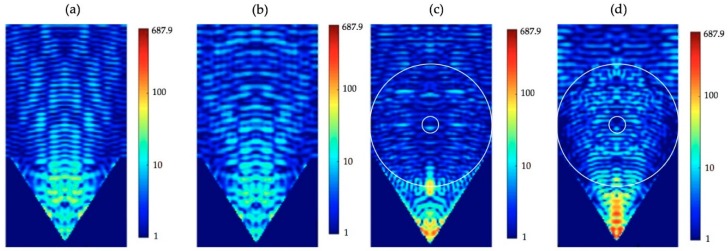
Visualizations of the FDTD-computed electric field intensities of (**a**) Klarite substrate only in water, (**b**) Klarite substrate only in air, (**c**) Klarite substrate with the core-shell microspheres in water, and (**d**) Klarite substrate with the core-shell microspheres in air. To ease visualization, (**a**–**d**) are in logarithmic scale and limited from 0 to 687.9. The maximums of the electric field intensities in (**a**–**d**) are 94.8, 66.3, 451.2, and 687.9, respectively.

The materials used for the design for the core-shell microspheres are hydrogel polymers, which have great transmittance in the visible light and high water content with additional advantages of being hydrophilic and most importantly biocompatible. Typically, hydrogel polymers absorb a significant amount of water. Therefore, our hydrogel core-shell microspheres can be useful for the applications in the biological aqueous environments. To demonstrate the usage of these hydrogel core-shell microspheres, we took our designed core-shell microspheres in an experiment for improving the SERS technique. The SERS technique is a popular method to enhance Raman signals which can be used for the detection of biological or chemical molecules [47,48]. The plasmonic micro-nanostructures are often used to increase Raman signals by the plasmonic effects. For example, the tips with a controlled size inside a Klarite substrate can be used to further enhance the Raman intensity [49]. The Klarite substrate is a standard commercially available substrate for SERS application. It is an inverted pyramidal nanostructure with a gold overcoat on its surface. Here, we took the Klarite substrate as our SERS underlying substrate and compare the simulation results with and without putting the additional core-shell microspheres on the substrate. A schematic diagram for our simulations of core-shell microspheres placed on Klarite substrate is shown in Figure 10a. The cross sectional structure of the setup is also shown in Figure 10b. For the refractive index of the silicon, we used the experimental data from Palik’s optical handbook [50]. The refractive index of the silicon at 589 nm is 3.9725 + i 0.03 after fitting the experimental data. For the refractive index of the gold, we used the experimental data by Johnson and Christy [51]. The refractive index of the gold at 589 nm was 0.2713 + i 2.9516 after fitting the experimental data by Drude model. The fitting processes in Lumerical software were used to set the parameters and weights to fit the experimental data [52]. This kind of structure can be fabricated by the sphere lithography technique. For example, several different periodic micro-nanostructures can be fabricated by using this technique for the surface-enhanced Raman spectroscopy [53]. The corresponding three-dimensional (3D) domain for the simulation was set up as 6 µm × 6 µm × 19.5 µm and the spacing of a uniform mesh was 10 nm to ensure that the simulation results can be numerically converged in the reasonable numerical errors. The inverted pyramidal structure can be fabricated by mask patterning and etching process [49]. For the <100>-oriented silicon wafer, the angle between the faces of the inverted pyramids and the flat surface of silicon was 54.74° and the angle between the edges of the inverted pyramids and the flat surface of silicon was 45°. Figure 11a,b show the electric field intensities of the Klarite substrate in water and in air without the core-shell microspheres, respectively. The enhancement of the electric field intensity due to the surface plasmon resonance was found to be about 94.8 folds and 66.3 folds in water and in air, respectively, in the area a little bit away from the pits of the inverted pyramidal structure. The core-shell microspheres have the ability to modulate the focal distance of the generated photonic nanojets. The core-shell microspheres can be placed in a position at the void of the Klarite substrate where strong intensity of the photonic nanojets can be created. Figure 11c shows the electric field enhancement for the case in which core-shell microspheres are placed above on the Klarite substrate in water, where $n_{s}$, $n_{1}$, $n_{2}$, $r_{1}$, $t/r_{1}$, and λ are 1.33 (water), 1.4232, 1.3640, 3 μm, 13/15, and 589 nm, respectively. It can be seen that adding the core-shell microspheres can enhance the electric field intensity from 94.8 to 451.2 folds. The surface enhancement Raman scattering is related to the fourth power of electric field magnitude, i.e., the electric field intensity to the second power. The core-shell microsphere placed above a Klarite substrate in water with schematic diagram shown in Figure 11c can be used to enhance the Raman signal by 23 times compared with the case shown in Figure 11a where no core-shell microspheres are present. The electric field intensity in the case with core-shell microspheres above the Klarite substrate in water did not enhance much because the focal distance of the photonic nanojet in water was much longer than the depth of the void of the Klarite substrate. As described in Section 3.3, it was found that the generated photonic nanojets by core-shell microspheres in air had a shorter focal distance and a stronger intensity than those from the case in water. Figure 11d shows core-shell microspheres above the Klarite substrate in air. It was found that the electric field intensity was enhanced from 66.3 to 687.9 folds. This means that the Raman signals of the case shown in Figure 11d can be enhanced by about 108 times compared with the case in which no core-shell microspheres are placed on top of the Klarite substrate in air (Figure 11b). Therefore, the core-shell biocompatible microspheres with a careful design can have great potential in biosensing applications.

## 4. Conclusions

The photonic nanojets that are generated by the core-shell microspheres made from novel biocompatible hydrogel materials have been studied numerically. In particular, the guidelines to generate narrow, long effective length, and high intensity photonic nanojets are given. Two kinds of biocompatible materials P407DA/PAA and P407DA/PHEMA have been used for the design of the core-shell microspheres. Thus, the characteristics of the photonic nanojets constructed, such as the focal distance, intensity, effective length, and FWHM, can be adjusted by varying the thickness of the shell layer in both high-core and low-core core-shell microspheres. The overall size of the core-shell microspheres can also affect the photonic nanojets. It was found that for larger core-shell microspheres, the focal distance became longer, the intensity grew stronger, the effective length became longer, and the FWHM got wider. The surrounding medium also played an important role in generating photonic nanojets from the core-shell microspheres. The produced photonic nanojets exhibited longer tails in water than the core-shell microspheres that were placed in air. However, the core-shell microsphere in air showed stronger electric field enhancements than the particle immersed in the water. To demonstrate their usage, we applied the photonic nanojet producing biocompatible core-shell microspheres to enhance the SERS signal on a Klarite substrate. Compared with the results of the Klarite substrate in water and in air without the core-shell microspheres, the Raman signals produced from core-shell microspheres can be significantly enhanced about 23 times in water and 108 times in air. These results suggest that the biocompatible core-shell microspheres can be useful to be employed in future biosensing applications.

## Figures and Tables

**Table 1:** Intensity, effective length, focal distance, and FWHM in surrounding mediums of water and air. FOM: figure of merit.

Medium	Intensity	Effective Length (λ)	Focal Distance (λ)	FWHM (λ)	FOM
water	38.87	14.47	13.36	1.307	430.4
air	147.55	1.76	1.03	0.734	353.8
